# American Medical Association, Section on Stomatology

**Published:** 1904-05

**Authors:** 


### AMERICAN MEDICAL ASSOCIATION, SECTION ON STOMATOLOGY.

(Concluded from page 217.)

### FOURTH SESSION.

THE meeting was called to order at 2.10 P.M., with Dr. M. H. Fletcher, of Cincinnati, in the Chair.

### DISCUSSION ON DR. WILLIAM E. WALKER'S PAPER ON "ORTHODONTIC FACIAL ORTHOMORPHIA."

(For Dr. Walker's paper, see page 193.)

*Dr. Eugene S. Talbot.*—It is very necessary that we should understand the etiology in order to treat these cases successfully. It is unfortunate that in this country the majority of the men treating these cases are mechanics, not pretending to know anything about the etiology. Indeed, one of the best men in the country in this line of work, says he does not care for the etiology, that it is enough for him to be able to treat the conditions. I was surprised and pleased when I went into Dr. Walker's office to find that he grasped the essential idea. It requires a great deal of time and study to understand the manner in which these conditions are produced, and in order to study this one particular phase we must go back to the time of Aristotle. Aristotle laid down the law of economy of growth, whereby the structure or organ is lost for the benefit of the organism as a whole. All students in evolution have based their work on that law. I know of no better work on this subject than "From the Greeks to Darwin." This law of economy of growth is illustrated in many structures, but those of the face, jaws, and teeth are the most important to us. They are being lost for the benefit of the brain. That is the reason we have so much decay in the teeth. That is the reason the jaws grow smaller and the teeth, while they do not grow smaller, work in harmony with this arrest of development. Because of this arrest of development of the jaws and because the great diameter of the teeth is larger than the jaws, we have this arrest of development and irregularities. In the evolution of man we also have atavism, so that in these patients which we call neurasthenic and which I call degenerates, where there is an arrest of development, there is also frequently excessive development. These are beautifully illustrated in two pictures which I shall give to you. One is of Judas. If the painter lived at the present time he could not have portrayed a better representation of a degenerate than he has done. The second picture is that of Charles V. of Spain. Those of you familiar with the history will recall that Charles V. suffered with indigestion all his life; that he could not eat, and that he kept ten cooks preparing food for him. Two hundred years after his death a photograph was taken of his skeleton in the coffin, showing this type of face. With the mouth closed the lower jaw shut above the upper. In other words, the upper teeth closed inside the lower jaw. We had no Walkers at that time, and therefore he could not have his teeth regulated. The lower jaw is a movable organ, and because of this it is frequently more fully developed when the upper jaw, which is attached to the bones of the skull, becomes arrested in development, and hence we have this marked deformity. It is possible that the lower jaw in some cases will be excessively developed and there atavism enters. If such cases are taken at the age of thirteen when the alveolar process is in construction there is a possibility of moving these teeth and having the alveolar process remain with them. If the correction is made at the age of twenty to twenty-four, there is more motion, producing inflammation in the alveolar process and preventing its restoration. Therefore, I question how far we, as operators, are justified in correcting irregularities of the teeth. Every patient coming to us like this little one referred to is a degenerate, having an unstable nervous system. When we undertake to regulate these teeth we put a pressure upon the nervous system of that child which is a very serious matter. I have known many cases laid up from one to three years in bed from nervous prostration as a result of dentists regulating these teeth and not knowing when they were producing neurasthenics. I do not believe that the ordinary dentist should undertake to treat these cases, but one who is medically educated and who is familiar with the general nervous system. I shall look with great interest and pleasure for the results in these cases Dr. Walker has in hand, and which he is so well able to treat.

*Mr. T. E. Constant.*—I should like to congratulate Dr. Walker upon the method he has pursued in the correction of the deformity, and I can quite concur with him in what he says with regard to the necessity of studying his cases. Very often in the condition where the upper incisor teeth apear to protrude we find that a study of the etiology results in showing a still greater deformity. In many cases diagnosed as superior protrusion the condition is really one of inferior retrusion. This is admirably represented in one case, and I am sorry the patient is not here.

I quite disagree with Dr. Talbot in what he says regarding the moving of the teeth. I think there is no reason, if a good bite is obtained after the teeth have been removed, why the result should not be permanent. The only thing I fear is that it would be extremely difficult to obtain a bite that would be permanent. That will depend entirely upon the condition with which the teeth occlude when the case is finished.

*Dr. M. H. Fletcher.*—I have nothing to say except in commendation of the essayist from the stand-point he takes, which would indicate his ability to cope with the case. I fully concur with Dr. Talbot in his condemnation of the efforts of incompetent men to do this work. Personally, I have had three cases sent to me in a state of collapse almost from ineffectual efforts of men who apparently understand nothing about the work or had no conception of the stress to the patient. The cases resulted in an absolute failure and a good wholesome fear on the part of the patient of everybody who would undertake the work. These cases have made me a little more timid in my own efforts. The position of the patient and the varying conditions of health, also the surroundings at home have great bearing upon these cases. I see that the child has absolute co-operation and encouragement of the home people. I have had several cases in which there was a tendency to make light of and poke fun at the patient. I absolutely demand that such things shall stop. Otherwise I give up the case. I am to have the absolute co-operation of every one of the family, and I

find that better progress is made in this way. The child must have normal recreation. My plan is never to proceed with any painful measure until restoration of the nervous system is complete. As Dr. Talbot has said, it is a pleasure to see these cases presented by Dr. Walker, who is able to carry them to a successful end.

*Dr. H. E. Belden,* New Orleans.—I agree with Dr. Talbot that the operation carried on in adult life does not always meet with success, especially where a good deal of motion is acquired. I had a case of a young lady whose teeth protruded a great deal. I brought the teeth back to their place, but she has to wear a band to this day. I agree also with Mr. Constant that if the occlusion is good we are more apt to have a successful issue. We see many bridges which, because of inaccurate occlusion, patients are unable to wear.

*Dr. Walker.*—Showing how little the treatment interfered with this man's nervous condition, I may remark that he took during the treatment two years' course of law in one year and graduated. He has not yet secured occlusion. This we hope to obtain later. The patient tells us that he had not much pain, a little at the first. The bands worn were always stationary.

*Dr. M. L. Rhein.*—I was very much pleased to hear the remarks of Dr. Talbot in the discussion of this subject. His words almost exactly expressed my views. I think it fortunate that he should have brought out this point of criticism against men doing work of this nature strictly from a mechanical point without regard to the etiological factors at work. In this country of ours it is unfortunate that a certain class of men get a pseudo sort of dental education, with some mechanical dexterity, and appear before the public as advertised specialists on orthodontia. Personally I am a believer in the specialty of stomatology as a specialty of medicine, but I am opposed to any further subdivision of the work. I believe that the stomatologist should have the entire care of the individual mouth under his charge, and if the distinctive and individual portions of the work are to be divided up for the better method of execution, it all ought to remain under his personal supervision. There are a few exceptions, men who have the proper education back of their ability to handle the work. The amount of damage that is done by men improperly qualified is very great. It is my opinion that work cannot be commenced too early in these cases of irregularity. The same principle is brought out here as in the work of Lorenz. The softer the bones are when we handle them, the more easily are they moulded and the better are the results. I have attempted a number of corrections after the age of twenty, and the results have never come up to my hope. It seems almost impossible to get occlusion that is what might be called self-locking. I do not wish to decry the work that can be done at a certain age, but to say that the best results are obtained in the early work. I have at present two patients, one of the age of eight and the other of nine. There was much question in the mind of the parent whether to have work commenced at the time, and the opinion of a number of men was sought. A large number of the profession have the idea that work should be delayed until all the permanent teeth are erupted.

I take exception to the view that the permanent apparatus is less painful than the temporary. There is more than one way of applying one apparatus. I know of no apparatus that will work so painlessly, so easily, so rapidly without the patient paying attention to it as that devised by Dr. Ottolengui. It has only recently been described by him, although it has been in use for some time. I agree that if the work upon the bones cannot be done without giving pain and causing nervous tension, there is a mistake somewhere, and that much harm will be done the child under such conditions. These patients come to my office sometimes as often as four times a week without any evidence of nervous depression. I claim that that is an absolute necessity if the work is to be done at the early age in which the best results can be obtained.

*Dr. Walker* (closing).—I feel very much pleased with the way in which the presentation has been received, but I must disagree on some of the points. Dr. Talbot, for instance, I think made a reflection upon the effect of this work upon neurasthenics. The child referred to is a most decided neurasthenic, but since I have been working upon her teeth her health has been manifestly improved daily. As the treatment continued, her mental, physical, and nervous condition became better. I think this has been partly due to the self-control I have caused her to exert. Heretofore, the least disturbance would cause her to cry.

The Chairman has stated that the work should be practically painless. He is undoubtedly right in that. The old way of regulating by movable appliances did cause a great deal of pain and damage to the nervous system. With the fixed appliance, however, the soreness does not ensue. I have used Jackson's appliance for some time. It is the one to use when the teeth are coming in. If the teeth are in place the fixed apparatus is better because a greater force is required than can be secured with the Jackson appliance without producing soreness. I agree with the Chairman that the work should be done while the teeth are coming in.

I think no one system should be adhered to, but that the good of all should be selected for the case to which it is adapted. We should undoubtedly avoid producing pain on account of the injury to the nervous system. In cases in which not much pressure or a great degree of speed is needed I feel that a movable apparatus is the one to employ.

Mr. Constant is correct in saying that many cases of inferior retrusion are diagnosed as superior protrusion and so treated and the patients disfigured.

In regard to specialization, I do not by any means think that a man should practice orthodontia without previous general practice, but I think more will be accomplished by a man taking up a specialty after a number of years spent in general practice, and then working in sympathy with the general practitioner.

A paper was read by Dr. George F. Eames, of Boston, entitled "The Therapeutic Use of the X-Ray in the Oral Cavity."

(For Dr. Eames's paper, see page 356.)

DISCUSSION.

*Dr. Eugene S. Talbot.*—I can say very little on this subject, because I have had no experience in this line. I have heard that Dr. Price, of Cleveland, is doing some remarkable work in interstitial gingivitis with this apparatus. From what the essayist has said, and from what I have heard from others, I see no reason why this should not do splendid work in this disease. It has not been tried long enough to give any definite results. It is barely possible that, because of the situation of the tissues inside the mouth, satisfactory results have not been obtained. I regret very much that I am not familiar with the subject and able to discuss it. I would like to ask Dr. Eames if the elimination of the oxygen is comparable to the effect produced in the two poles. I remember a demonstration that was made with a piece of meat in which the coagulation of albumin was made at the cathode pole. Do I understand that the action here is similar to that? From what does it eliminate the oxygen.

*Dr. Eames.*—The oxygen is liberated from the air which is contained in the tissues and whatever may be surrounding the tissues. It goes right through and decomposes everything with which it comes in contact.

*Dr. Fletcher.*—I suppose the rays can be seen coming out the other side, if examined with the fluoroscope?

*Dr. Eames.*—It penetrates the tissues depending upon the tube. The low vacuum or soft tube has greater power for destruction of tissues, but it does not penetrate so far. With surface conditions the soft tube is desirable, because it does greater work. In a growth in the stomach we would have to have a tube of higher power and the quality of the electricity changed greatly.

*Dr. Fletcher.*—In this case, do you have to use a soft tube?

*Dr. Eames.*—Yes, that penetrates deeply enough. The distance in the mouth does not count because we hold the tube near the face, so that practically a place in the mouth is on the surface.

*Dr. W. E. Walker,* New Orleans.—I have had no experience with the X-ray in therapeutic work. Dr. Woodward, of this city, tells me that he has secured very satisfactory results in Riggs's disease with the X-ray.

*Dr. Fletcher.*—Did he couple it with any other treatment?

*Dr. Walker.*—What I have told you is as much as he told me.

*Dr. Hans Pilcher,* Vienna.—I would like to ask Dr. Eames if there is any possibility of the X-ray having any influence upon calcareous pericementitis if it is not removed?

*Dr. Eames* (closing).—There is very little for me to say beyond what is contained in the paper. I might say, in addition to this paper, that the sun's rays have been utilized in a therapeutic way, and those who have used the actinic rays, so-called, from the sun have in a very simple and inexpensive manner achieved results in many cases similar to that in the use of the X-ray. It only waits for a trial to see whether the rays of the sun may be utilized in the mouth. The use of the sun's rays, of course, is limited to the time when the sun shines. That alone would indicate an opening for some one to utilize the rays for the therapeutic treatment of disease.

A paper entitled "Methods of controlling Hemorrhage of the Oral Cavity" was read by Dr. H. T. Belden, of New Orleans.

(For Dr. Belden's paper, see Vol. XXIV., page 502.)

DISCUSSION.

*Dr. M. L. Rhein.*—The use of adrenalin in dentistry and its advantage should be known to all of us. I simply echo its value as an important part of our pharmacopœia in dental work.

*Dr. George F. Eames.*—I have used the extract of the suprarenal glands and the various preparations made from it for some time, and I can only say that my experience with it has been very satisfactory. It is the most wonderful drug in the control of hemorrhage I have ever used. In some cases it does not seem to act, but these cases are so rare that they may be classed as cases of idiosyncrasy or peculiar unsusceptibility to the drug. I think Dr. Belden is correct in saying that it probably does enhance the effect of cocaine, but that alone it is not an anæsthetic.

The peculiar diathesis of a patient should be considered and the greatest caution used in the preparation of the patient for an operation which is apt to cause hemorrhage.

*Dr. M. L. Rhein.*—I do not agree with Drs. Belden and Eames in their views concerning the anæsthetic properties of adrenalin. I believe that we as stomatologists demand a much more profound anæsthesia than the laryngologist in his work. I do not believe it is possible to obtain profound anæsthesia—that is, local anæsthesia—with the chloride of adrenalin. Used in the nose or the eye, a certain amount of anæsthesia is unquestionably obtained, but the fact that it is not complete anæsthesia I think blinds us to the anæsthetic properties that it does possess. I think this anæsthetic property is produced mechanically by the contraction of the capillaries expelling the nerve-cells from the fibrillæ. The fact that we are not satisfied with its obtunding effect is not proof that it has not a certain amount of anæsthetic property.

The papers of Dr. Richard Grady, of Baltimore, upon "The Dentist in the United States Navy," and of Dr. Alice N. Steeves, of Boston, upon "Medical and Dental Libraries," were read by title.

